# Adding Strength to Strength

**Published:** 1883-12

**Authors:** 


### ADDING STRENGTH TO STRENGTH.

The *New England Journal of Dentistry,* which has heretofore been edited and published impersonally, in its last number announces its syndicate and publishes the names of its editors. It has a number of accessions to its corps of collaborators, and will enter upon its third volume with brighter prospects than ever. The INDEPENDENT PRACTITIONER acknowledges the compliment paid in adopting its method of publication, and wishes the *N. E. Jour.* abundant success. Good as it has always been in the past, we shall expect that it will be better in the future.

